# First confirmed case of monkeypox in Adamawa State, Nigeria: a clinico-epidemiological case report

**DOI:** 10.11604/pamj.2022.42.38.34715

**Published:** 2022-05-16

**Authors:** Emmanuel Pembi, Sati Awang, Saheed Olalekan Salaudeen, Innocent Adoyi Agaba, Semeeh Omoleke

**Affiliations:** 1 State Ministry of Health and Human Services, Yola, Adamawa State, Nigeria,; 2 Department of Internal Medicine, Federal Medical Centre, Yola., Adamawa State, Nigeria,; 3 23 Brigade Medical Centre, Yola, Adamawa State, Nigeria,; 4 Doctoral Programme in International Public Health, Euclid University, Bangui, Central African Republic

**Keywords:** Monkeypox, surveillance, Adamawa State, Nigeria, case report

## Abstract

Monkeypox is a rare zoonotic infection caused by monkeypox virus. It is an emerging disease which has become the most prevalent orthopoxvirus since the global eradication of smallpox in 1980. It is a mild illness which is mostly characterized by a prodromal of fever, malaise and progressive appearance of vesiculo-papular skin lesions. We report a case of a 30-year-old military personnel who was referred from 23 Brigade Medical Centre (BMC) to Federal Medical Centre (FMC) Yola on account of fever and progressive eruption of widespread skin rashes. Following isolation from suspicion of monkeypox, he was confirmed by PCR and managed symptomatically and fully recovered within two weeks of onset. His five close contacts did not develop any symptoms during the period of follow-up. This is the first confirmed case of monkey pox in the state. This case will trigger an awareness, amongst clinicians and surveillance officers in Adamawa State, of the existence of monkeypox and heighten the suspicion to promptly detect, isolate and treat more cases to halt transmission.

## Introduction

Monkeypox is a rare zoonotic infection caused by monkeypox virus [1]. It is an emerging disease that has become the most prevalent orthopoxvirus since the global eradication of smallpox in 1980 [2]. It was first reported in laboratory monkeys in the United States of America in 1958 and later in an infant from the Democratic Republic of Congo (DRC) in 1970 [3]. It is endemic in the DRC, but sporadic outbreaks occur in parts of Central and West Africa (Sierra Leone, Nigeria, Côte d´Ivoire), and the USA [4]. Since late 2016, there have been increasing reports of cases from African countries that have not reported cases for the past 40 years [5].

Monkeypox is a mild illness that is mostly characterized by a prodrome of fever, malaise and progressive appearance of vesiculo-papular skin lesions [4]. The symptoms are similar to but milder than smallpox in humans. It is often self-limiting, but the case-fatality rate can reach 10%, particularly in children [6]. The incubation period is 7-14 days but can range from 5-21 days [7]. It is transmitted to humans from rodents and primates, but human-to-human transmission also occurs through contact with lesions, body fluids, respiratory droplets and contaminated materials (e.g., bedding) [6]. Concern has recently been raised on the emergence of a severe clinical pattern of human monkeypox virus resembling smallpox [8]. There is also an increase in frequency and geographic spread across West and Central Africa in recent years [8]. For example, Nigeria reported 3 cases of human monkeypox between 1970 and 2017; one and two cases in 1970 and 1978, respectively [4]. However, in 2017, a re-emergence of the largest outbreak occurred in Nigeria with 228 suspected cases (60 confirmed cases) in 24 of the 36 States plus the Federal Capital Territory. Most cases reported were less than 40 years (born after the smallpox eradication campaign) and lacked cross-protective immunity [4]. Before now, Adamawa State has never reported a confirmed case of monkeypox. In this paper, we described the first case of monkeypox recorded in Adamawa State, Nigeria, and enumerated some good practices and challenges faced in responding to the identified case.

## Patient and observation

**Patient information**: we report a case of a 30-year-old military man who was referred from 23 BMC, Yola to FMC Yola on account of fever, progressive eruption of widespread skin rashes and sudden collapse. The patient was apparently well until a few days before his engagement in four days of intensive military drill. He started expressing some mild fever and tiredness about two days before the commencement of the drill. On the fourth day of the drill, he suddenly collapsed and was rushed by the military paramedics to the 23 Brigade Medical Centre (BMC), where he was observed to have pruritic rashes. The rashes were said to have first appeared on the forehead, groin and genitalia before progressing to his chest, back and periphery of the appendages, including the palmer and dorsal aspects of the feet and hands. He was assessed to have secondary syphilis following a positive VDRL and was subsequently referred to FMC Yola for expert care. The case was immediately isolated as a case of suspected monkeypox following history taking and clinical examination in the Infectious Diseases Unit, FMC Yola. Swab samples from lesions were collected and shipped to National Reference Laboratory (NRL), Abuja, for confirmation. Clinical history taken indicated no past history of rashes or consumption of medication. However, he had his first dose of the COVID-19 vaccine about a month before symptom onset. An inconsistent recent history was found regarding the possibility of travel and contact with the forest ecosystem. There was no history of contact with any person with such manifestation within the past three weeks before onset. However, he was in the bush for two years and returned home six months prior to symptom onset. Since his arrival home, the patient had not been to the bush or traveled outside the town. There was no positive history of consumption of rodents, bush meat, or hunting for animal protein. The patient has been married but partially separated from his wife for about a year. He had not received the smallpox vaccine.

**Clinical findings**: on examination, the patient is a strong and agile young man, well-fed, fully alert, conscious, and well oriented in time, place and person. He was afebrile (36.2ºC), anicteric, acyanosed, and well-hydrated with globalized vesiculo-papular skin rashes. No associated peripheral pedal edema.

**Figure 1:**
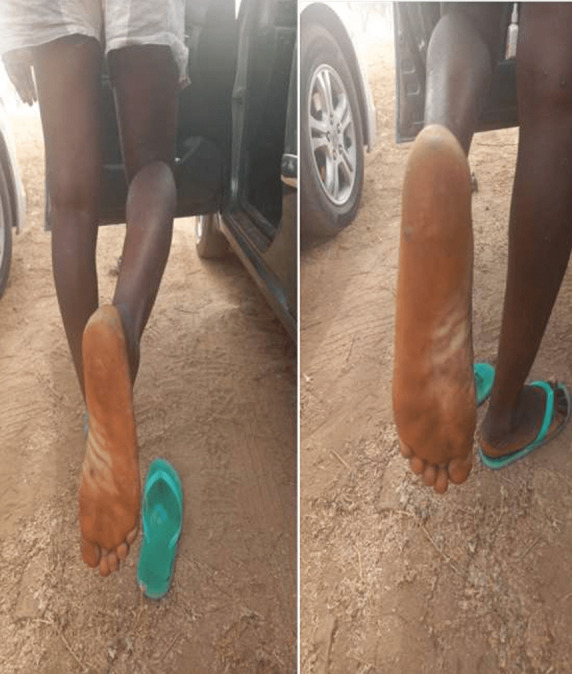
monkeypox lesions on both soles

**Figure 2:**
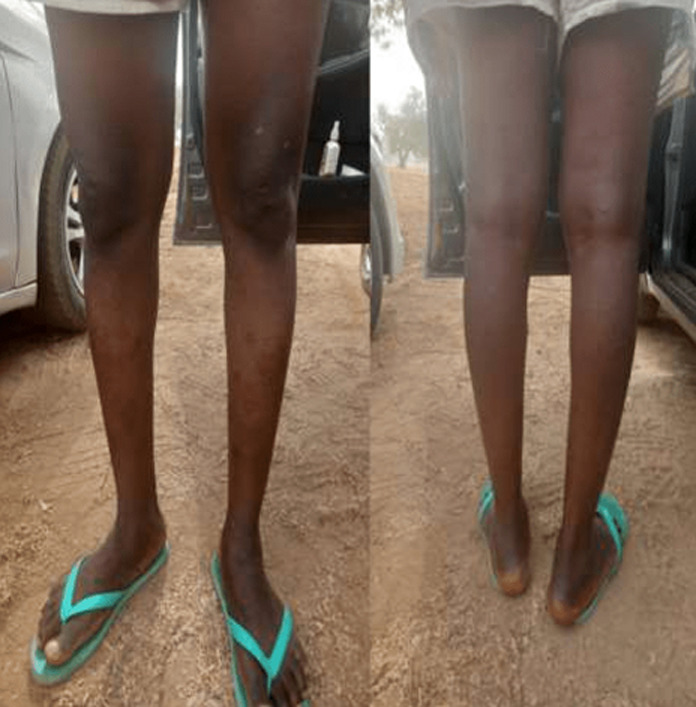
monkeypox lesions on both legs

**Figure 3:**
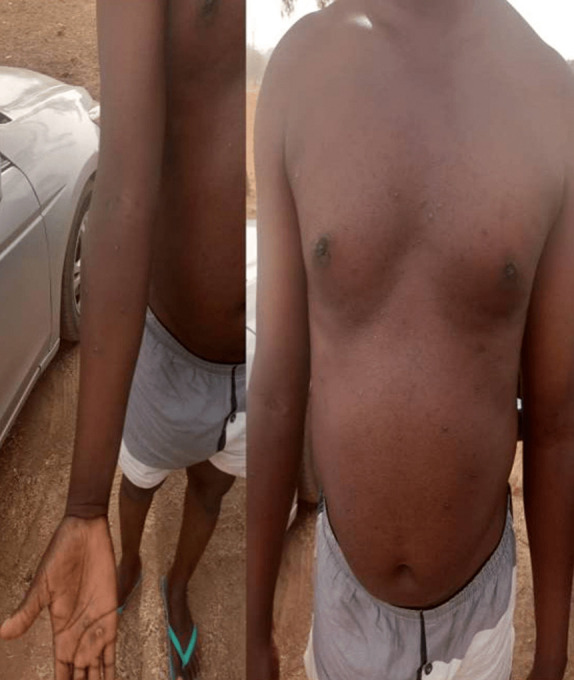
monkeypox lesions on the chest and hands

**Status localis**: widespread vesiculo-papular rashes involving the face, head, neck, trunk, buttocks, upper and lower extremities and the genitalia were found (Figure 1, Figure 2, Figure 3). There were bilateral enlarged cervical and inguinal lymph nodes, which were firm, non-fluctuant and tender. Other systemic examinations were not contributory.

**Diagnostic assessment**: laboratory investigations, including FBC, ESR, LFT, EUC and urinalysis were requested and found within normal ranges. He was counseled on care and isolation. His sample was collected and shipped to Nigeria Center for Disease Control, which processed the sample and released the result as positive for monkeypox by PCR.

**Diagnosis**: suspected monkeypox and later confirmed as monkeypox by PCR.

**Therapeutic interventions**: on admission to the isolation ward of the Federal Medical Centre, he was started on antibiotics (Ampiclox) to prevent secondary bacterial infection, paracetamol for pain management, vitamin c to enhance wound healing and artemether combination therapy for malaria.

**Follow-up and outcome of interventions**: a week after the onset of illness, an epidemiological investigation was launched by a multisectoral state team comprising the State Ministry of Health staff, Local Government Area (LGA) staff, staff of NCDC and the WHO staff to conduct risk and need assessment and to identify additional cases who had contact with the index case. The joint investigation team identified five contacts (two health care workers and three family members). All the identified contacts showed no symptoms or signs of monkeypox following their period of isolation. Like the index case, none of the contacts had any prior vaccination against smallpox.

**Patient perspective**: initially, the patient was shocked to have monkeypox because it is not a known disease (not common in this setting) and wondered how he contracted it. He was, however, happy to have recovered within two weeks.

**Informed consent**: the patient consented to his clinical information except name to be published to contribute to science and global health. He also consented to his picture being used without showing his face to avoid potential stigmatization and maintain privacy.

## Discussion

This is the first case of monkeypox ever reported in Adamawa State. Monkeypox is a re-emerging viral zoonotic infection that has become more prevalent after the eradication of smallpox in 1980 due to the out phasing of the smallpox vaccine, which offered cross-protective immunity against monkeypox. Between 2017 and 2021, Nigeria reported 446 suspected cases and 199 confirmed cases from 18 States. Almost all these cases occurred in persons less than 40 years of age as this cohort lacks cross-protective immunity against monkeypox which the smallpox vaccine offers older age group that was vaccinated against smallpox. The case in Adamawa is 30 years of age in keeping with the occurrence of monkeypox in those ages less than 40 years. This can guide policymakers in utilizing the limited resources available by prioritizing vaccines (such as the existing smallpox vaccine, JYNNEOS- a refined smallpox-monkey pox vaccine approved by FDA) for populations younger than 40 years who are at a relatively increased risk compared to older age group with cross-protective immunity against monkeypox.

The patient was also found reactive for syphilis when evaluated at the 23 BMC Yola before his referral to FMC Yola on account of secondary syphilis. Syphilis mimics monkeypox in its clinical manifestation of fever, sore throat, headaches, swollen lymph glands, muscle aches, skin rashes and/or mucous membrane lesions. However, patchy hair loss, painless chancre and weight loss that often occurs in syphilis are not seen in monkeypox. Other differentials include chickenpox, measles and scabies. Although chickenpox rashes resemble monkeypox in distribution over the body, the rashes are often in different stages of development, unlike those found in monkeypox, which is often in the same stage of development (no change in appearance once erupted). Additionally, in chickenpox, pustules and lymphadenopathy are not usual manifestations. This is contrary to manifestations in monkeypox, in which pustules and lymphadenopathy are a common occurrence. Scabies is different from monkeypox by its characteristic intense itching and onset of a pimple-like itchy rash affecting the wrist, elbow, armpit, webbing between the fingers, nipple, penis, waist, belt-line, and buttocks. Tiny burrows caused by the female mite are also often seen on the skin in scabies. These features are absent in monkeypox and were not found in this patient. Measles was ruled out easily as the patient lacked high fever, cough, coryza, and conjunctivitis, which are classical manifestations of measles. In measles, the rashes are maculopapular, while Koplik spots may also appear inside the mouth 2-3 days after symptoms in measles. These manifestations were absent in this patient.

Monkeypox is usually associated with direct or indirect contact with infected wild animals or humans. This is often found in the history of preparation and consumption of wild animals, history of jungle/bush visit(s), and history of travels to areas where transmission of monkeypox is ongoing. The patient declined any such exposures during posting or off duty within three weeks preceding the onset of his clinical symptoms. He also denied any contact with wild animals either through consumption, preparation, or visit by friends or families from other areas known to be in active transmission. There may be contact with sub-clinically infected individuals or through other social activities that the patient could not recall, or the case has deliberately concealed vital information related to his exposure to monkeypox for some reason(s). This may be the case, considering the allegation that might follow if a serviceman is found to travel from his duty location without permission, especially in North-East Nigeria, where insurgency and banditry are prevalent. Although this is our first-ever reported confirmed case, there had been previous reports of suspected cases of monkeypox in Adamawa State. Those suspected cases were discarded after laboratory investigation and were found negative for the disease. This indicates some level of suspicion/sensitivity of the existing surveillance system. The well-structured surveillance system in the State was able to promptly detect the case and immediately report the case-based information to appropriate levels. This immediately triggered a well-coordinated outbreak response. The patient was immediately isolated, sample/specimen was aseptically collected and sent under strict safety conditions to NRL through Tranex (a designated logistic firm) to confirm the diagnosis. Supportive and symptomatic treatment was commenced under strict infection prevention and control (direct contact and airborne infection prevention control measures).

The military barracks also intensified its infection prevention control activities and suspended some of its activities which had the potential to increase the crowding of military men. The Emergency Operations Centres for cholera and COVID-19 in the State were notified and instructed to integrate risk communication and community engagement regarding monkeypox into the existing activities to boost prevention, early detection and reporting while intensifying active case search and contact tracing to identify more cases. The intensive active case search and contact tracing identified five contacts who were line-listed and monitored with no symptoms detected during their follow-up. Despite a concerted effort by the State, FMC, WHO, NCDC and the military, some setbacks and challenges were identified in the response. First, after suspecting and confirming the case, none of the health personnel that attended to the patient has been vaccinated with the monkeypox vaccine and no effort has been made to secure this. Secondly, there are no isolation wards to cater for monkeypox in case of outbreaks in other LGAs. Thirdly, the suspicion of monkeypox was missed at the first point of contact until the patient presented to a Consultant Physician (Infectious Disease Specialist) at the FMC Yola, revealing the rarity and knowledge gap on monkeypox amongst healthcare professionals, especially at the primary healthcare level. We are unable to confidently link this case with any exposure due to an “inconsistent” history of contact with animals or visit to the bush provided by the patient. There may be a likelihood of concealment of “true” detailed history of exposure. This scenario further underscored the relevance of laboratory surveillance in disease detection and control.

## Conclusion

Monkeypox may be circulating in Adamawa State. The case report revealed some gaps in knowledge of uncommon tropical and infectious diseases by clinicians at the first point of care (primary care). Hence, the need to strengthen surveillance systems by training health workers and clinicians at the primary care level on emerging tropical infectious diseases, such as monkeypox.
